# Transgenic GDNF Positively Influences Proliferation, Differentiation, Maturation and Survival of Motor Neurons Produced from Mouse Embryonic Stem Cells

**DOI:** 10.3389/fncel.2016.00217

**Published:** 2016-09-12

**Authors:** Daniel Cortés, Yolanda Robledo-Arratia, Ricardo Hernández-Martínez, Itzel Escobedo-Ávila, José Bargas, Iván Velasco

**Affiliations:** ^1^Instituto de Fisiología Celular—Neurociencias, Universidad Nacional Autónoma de MéxicoMexico City, Mexico; ^2^Laboratorio de Reprogramación Celular del Instituto de Fisiología Celular, Universidad Nacional Autónoma de México en el Instituto Nacional de Neurología y Neurología “Manuel Velasco Suárez”Mexico City, Mexico

**Keywords:** glial cell line-derived neurotrophic factor, committed neural precursors, spinal motor neuron, electrophysiological maturation, excitotoxicity, neuroprotection

## Abstract

Embryonic stem cells (ESC) are pluripotent and thus can differentiate into every cell type present in the body. Directed differentiation into motor neurons (MNs) has been described for pluripotent cells. Although neurotrophic factors promote neuronal survival, their role in neuronal commitment is elusive. Here, we developed double-transgenic lines of mouse ESC (mESC) that constitutively produce glial cell line-derived neurotrophic factor (GDNF) and also contain a GFP reporter, driven by HB9, which is expressed only by postmitotic MNs. After lentiviral transduction, ESC lines integrated and expressed the human GDNF (hGDNF) gene without altering pluripotency markers before differentiation. Further, GDNF-ESC showed significantly higher spontaneous release of this neurotrophin to the medium, when compared to controls. To study MN induction, control and GDNF cell lines were grown as embryoid bodies and stimulated with retinoic acid and Sonic Hedgehog. In GDNF-overexpressing cells, a significant increase of proliferative Olig2+ precursors, which are specified as spinal MNs, was found. Accordingly, GDNF increases the yield of cells with the pan motor neuronal markers HB9, monitored by GFP expression, and Isl1. At terminal differentiation, almost all differentiated neurons express phenotypic markers of MNs in GDNF cultures, with lower proportions in control cells. To test if the effects of GDNF were present at early differentiation stages, exogenous recombinant hGDNF was added to control ESC, also resulting in enhanced MN differentiation. This effect was abolished by the co-addition of neutralizing anti-GDNF antibodies, strongly suggesting that differentiating ESC are responsive to GDNF. Using the HB9::GFP reporter, MNs were selected for electrophysiological recordings. MNs differentiated from GDNF-ESC, compared to control MNs, showed greater electrophysiological maturation, characterized by increased numbers of evoked action potentials (APs), as well as by the appearance of rebound APs, sag inward rectification, spike frequency adaptation and spontaneous synaptic potentials. Upon challenge with kainate, GDNF-overexpressing cells are more resistant to excitotoxicity than control MNs. Together these data indicate that GDNF promotes proliferation of MN-committed precursors, promotes neuronal differentiation, enhances maturation, and confers neuroprotection. GDNF-expressing ESC can be useful in studies of development and disease.

## Introduction

Embryonic stem cells (ESC) are capable of self-renewal and differentiation towards any mature cell from the three germ layers. Neural induction of mouse ESC (mESC) has been used as a cellular model to understand basic mechanisms of differentiation and physiology of postmitotic neurons. Importantly, ESC have been differentiated using neural medium and stimulation with morphogens such as sonic hedgehog and retinoic acid for ventralization and caudalization of neural precursors, respectively; this protocol yields spinal motor neurons (MNs; Wichterle et al., 2002). Once differentiated, neurons should grow axons and survive in their environment. This survival has been linked to the stimulation of specific receptors by neurotrophic factors, such as glia cell line-derived neurotrophic factor (GDNF; Lin et al., 1993; Moore et al., 1996; Pichel et al., 1996). GDNF knock-out (KO) mice present alterations such as renal and uretal agenesis and absence of enteric nervous system. Other cell types including sensory, sympathetic and parasymphatetic neurons, as well as cranial (trigeminal) and spinal (L1–L6) motor nuclei are partially affected. This neurotrophin activates heteromeric receptor complexes formed by c-RET and GFRα1; reported mutations on both c-RET and GDNF are associated to neuropathological conditions such as aganglionar megacolon (Hirschprung’s disease; Angrist et al., 1996; Moore et al., 1996; Pichel et al., 1996). The effects of GDNF were first observed when it was applied to mesencephalic dopaminergic neurons, causing increased axonal branching and survival (Lin et al., 1993). Stimulation of GDNF receptors is also related to higher survival of MNs both *in vitro* and *in vivo* (Moore et al., 1996; Rakowicz et al., 2002). *In ovo* administration of GDNF rescues MN from programmed cell death during chick development (Oppenheim et al., 1995). Similarly, this neurotrophin is protective in a postnatal mouse axonal injury model (Oppenheim et al., 1995), and also rescues MNs in models of Amyotrophic Lateral Sclerosis (Krakora et al., 2013; Islamov et al., 2015).

GDNF also induces axonal sprouting (Mount et al., 1995; Rosenblad et al., 2000) and enhances the spontaneous neurotransmitter release in nerve-muscle co-cultures (Yang and Nelson, 2004). The properties that GDNF promotes in final differentiation and specifically in synaptic transmission and electrophysiological activity have been studied in *Xenopus laevis* where GDNF enhances spontaneous transmission and also an increase in quantal size (Wang et al., 2001, 2002). This initial electrophysiological characterization on the effects of GDNF was carried out in non-mammalian cells. When MN development has been studied in mammalian newborn MN, specific features of these neurons have been uncovered. Calcium and/or sodium channels appear early, while calcium-dependent potassium channels are expressed later, contributing to a rapid hyperpolarization and modulating firing rate (Spitzer et al., 2000). On the other hand, some of the electrophysiological properties in mESC-derived MNs have been characterized. These neurons present tetrodotoxin (TTX)-sensitive sodium currents, potassium conductance and nickel-sensitive calcium channels. In addition, they show action potentials (APs) or rebound APs (RAPs), after hyperpolarization, as well as spike frequency adaptation (SFA) but only after extended periods of time or after co-culture with muscle cells (Miles et al., 2004; Takazawa et al., 2012). Alternatively, the electrophysiological maturation of ESC-derived MNs can be achieved by co-culturing them with astrocytes (Bryson et al., 2014; Kiskinis et al., 2014) or primary neurons (Li et al., 2015) for long periods. Thus, maturation of MNs differentiated *in vitro* requires exogenous factors such as neurotrophins and/or the co-culture with other cell types; in these papers, no direct assessment of GDNF actions on these electrophysiological properties were studied in ESC-derived MN.

Furthermore, a large amount of knowledge has been accumulated regarding the effects of GDNF in neuronal survival and some in maturation. However, very little is known about its role when neural precursors are still proliferating. For instance, research looking into the localization of GDNF and its receptors during development found that spinal cord, brainstem and dorsal root ganglia expressed GFRα1 from E10, when the neural tube has barely formed and neural precursors are not specified yet (Trupp et al., 1995; Treanor et al., 1996; Golden et al., 1999; Homma et al., 2000). At these early stages neuronal differentiation is starting while GDNF is expressed in the incipient limb bud, too early to induce any sprouting or trophic effects on postmitotic neurons. The fact that skeletal muscle appears after E12 to protract MN axons, suggests that GDNF might influence neural precursors.

The objective of the present set of experiments was to perform neuronal differentiation of ESC to MNs and to gain further knowledge on the role of this neurotrophin during neuronal differentiation, maturation and survival. Previously, we reported that mESC differentiate to MNs after stimulation of embryoid bodies (EBs) with retinoic acid and Sonic Hedgehog, producing MN positive for Beta-Tubulin III, GFP (reflecting Hb9 expression) and Islet1 (López-González et al., 2009). We now report the generation of mESC lines that constitutively secrete GDNF, from the pluripotent state to the terminal differentiated MNs. These engineered cells produced higher numbers of cycling neural precursors specified for MN, resulting in a higher proportion of terminally differentiated neurons. Using a MN-fluorescent reporter we performed electrical recordings. GDNF-expressing neurons exhibited more signs of maturity than control cells after 9 days of differentiation, without co-culturing them with muscle, astrocytic or neuronal cells. Furthermore, GDNF protected MNs from toxic insults such as kainate.

## Materials and Methods

### Lentiviral Construction

All recombinant DNA work was performed according to the National Institutes of Health Guidelines for Research Involving Recombinant DNA Molecules. Human GDNF (hGDNF) complementary DNA (cDNA) was a kind gift from Dr. Martha C. Bohn. The plasmid containing the GDNF cDNA (pAAV-MCS-hGDNF) was digested and the fragment containing the cDNA was separated by agarose electrophoresis and column-purified. The 639 bp fragment was ligated into the lentiviral vector pLVX-EF1α-IRES-Puro (Clonthech 631988) to produce the pLVX-EF1α-hGDNF-IRES-Puro plasmid. The resulting plasmid was sequenced and verified to contain hGDNF.

### Production of CTRL-ESC and GDNF-ESC

Lentiviral particles were produced accordingly to the manufacturer’s instructions (Clontech protocol PT5135-1). Empty vector (control, CTRL) or hGDNF containing plasmids, together with lenti-X HTC packaging mix and Xfect reaction buffer were mixed with Xfect polymer and then poured into a previously seeded HEK294FT cells. Forty-eight hours later, supernatants were collected and added to the R1 mESC. Puromycin treatment (2 μg/ml) was initiated 24 h later, and 4 days after treatment, several resistant colonies were obtained.

### Genotyping

Selected colonies were lysed and genomic DNA was extracted; end-point PCR for hGDNF detection was performed using a 30-cycle program at 59°C. Primers (all in 5′→3′) for GAPDH were: Forward, ATCACCATCTTCCAGGAGCG and Reverse, CCTGCTTCACCACCTTCTTG and for transgenic hGDNF: Forward, AACAAATGGCAGTGCTTCCT and Reverse, AGCCGCTGCAGTACCTAAAA. The same protocol and primers were used for mRNA using Trizol extraction protocol after DNase treatment (López-González et al., 2011; Rodríguez-Martínez et al., 2012).

### Western Blot

Briefly, cells were homogenized in lysis buffer (25 mM Tris–HCl pH 7, 1% IGEPAL, 100 mM NaCl) and supplemented with protease inhibitors (Roche). Proteins were obtained by centrifugation at 13,800 g at 4°C for 15 min, and quantified by Bradford assay (Bio-Rad). Proteins (30 μg) were resolved on 8% sodium dodecyl sulfate–polyacrylamide gel electrophoresis and transferred to nitrocellulose membranes (Amersham Bioscience). The membranes were blocked with 5% non-fat milk and incubated overnight with primary antibodies. Pre-stained markers (Invitrogen) were included for size determination. The following antibodies were used: rabbit anti-GDNF (1:1000, sc-9010 Santa Cruz Biotechnology) and anti-GAPDH (1:1000, MAB374 Millipore). Membranes were washed and incubated with corresponding horseradish peroxidase-coupled secondary antibodies (1:10,000, Santa Cruz) for 2 h. Immunoreactive bands were detected using the enhanced chemiluminescence method (Amersham Bioscience) and developed on photographic film (Kodak).

### Enzyme Linked ImmunoSorbent Assay (ELISA) for GDNF

To quantify secreted GDNF, 24 h-conditioned media were obtained from CTRL-ESC and GDNF-ESC. The media were collected from undifferentiated mESC and from day-6 EBs, which already contained differentiated MNs. The kit GDNF Emax Immunoassay system was purchased from Promega and used as indicated by the manufacturer’s booklet.

### Production of Reporter Lines to Monitor MN Differentiation

CTRL-ESC or GDNF-ESC were electroporated with HB9::GFP plasmid (a kind donation from Dr. Hynek Wichterle) and selected with neomycin (500 μg/ml) or hygromycin B (150 μg/ml), respectively, to create CTRL-ESC/HB9::GFP or GDNF-ESC/HB9::GFP.

### Motoneuron Differentiation

MNs were differentiated as reported (Wichterle et al., 2002) with some minor modifications. Briefly, ESC are maintained undifferentiated in KO DMEM supplemented with 15% fetal calf serum, 1% non-essential amino acids, 2 mM glutamax, 55 μM 2-mercaptoethanol, 50 UI/ml penicillin-streptomycin (all from Invitrogen) and 1000 U Leukemia Inhibitory Factor (ESGRO, Millipore). Cells were dissociated to form EBs in non-adherent plates in DFNK (DMEM:F12, Neurobasal, 10% serum replacement, 0.5 mM glutamax, 35 μM 2-mercaptoethanol and 50 UI/mL penicillin-streptomycin; Invitrogen) with medium change every other day. From day 2 to day 6 EBs are cultured in the presence of 2 μM retinoic acid and 10 μM purmorphamine (Millipore). At day 6 EBs are dissociated with TrypLE express (Invitrogen), seeded on poly-L-ornithine and fibronectin-coated coverslips in N2B27 (DMEM:F12, Neurobasal medium, N-2 supplement, and B-27 supplement plus penicillin-streptomycin; Invitrogen) for three additional days.

### Immunofluorescence

Cell cultures were fixed with 4% paraformaldehyde for 20 min and permeabilized with 0.3% Triton X-100 (Sigma). Primary antibodies were incubated overnight at 4°C in phosphate buffer saline (PBS) plus 10% normal goat serum. Cells were washed three times and secondary antibodies incubated for 2 h in the same solution, washed three times and incubated with Hoechst for nuclei staining. Primary antibodies were used with the following dilutions: Oct 3/4, 1:200 (BD 611202); Sox2, 1:1000 (Millipore AB5603); GFP, 1:1000 (Invitrogen A11122); Olig2, 1:1000 (Millipore MANB50); Islet1, 1:10 (developmental studies hybridoma bank 40.3A4-5 and 40.2D6); TUJ1 (recognizing Tubulin β III), 1:1000 (Covance MRB-435P); Microtubule Associated Protein (MAP2), 1:1000 (Chemicon MAB378); Phospho Histone H3, 1:100 (Cell Signaling 9701S). Alexa Fluor secondary antibodies were used at 1:1000. EBs cultured for 5 days were harvested by gravity, washed with PBS and paraformaldehyde-fixed, cryo-protected by sucrose, tissue tek-embedded, cut in 20 μm slices and mounted in slides for immunofluorescence protocol.

### Retro-Transcription Followed by Quantitative PCR

Total RNA was isolated from CTRL- and GDNF-ESC. Reverse transcription was performed with random hexamers to produce cDNA, as previously reported (Rodríguez-Martínez et al., 2012). The following primers were used to perform qPCR, starting with 100 ng of cDNA: *Oct4*: Forward (Fwd), TTG GGC TAG AGA AGG ATG TGG TT; Reverse (Rev), GGA AAA GGG ACT GAG TAG AGT GTG G. For *Sox2*: Fwd, GCA CAT GAA CGG CTG GAG CAA CG; Rev, TGC TGC GAG TAG GAC ATG CTG TAG G. For *Nanog*: Fwd, TAT CTG GTG AAC GCA TCT GG and Rev, GAA GTT ATG GAG CGG AGC AG. The house keeping gene *Actin* was used to normalize the expression of the former genes in CTRL-ESC HB9::GFP and compare them with GDNF-ESC HB9::GFP. Results are expressed as fold-change relative to CTRL-ESC.

### Electrophysiology

The expression of GFP under the HB9 promoter enables the visual identification of differentiated MNs. Because the main interest of several previous studies was the expression of the MN phenotype, such neurons were co-cultured with muscle fibers (Miles et al., 2004; Takazawa et al., 2012). However, this co-culture system might have uncontrolled factors in addition to GDNF. To test the actions of this single growth factor, we decided to study differentiated MNs without mixing them with muscle. Therefore, whole cell patch recordings on HB9::GFP-expressing neurons plated on fibronectin were performed after identification by fluorescence. Control and GDNF-expressing samples were recorded after 9 days *in vitro* in order to obtain signs of excitability in different cells. Together with different signs of excitability, membrane properties such as resting membrane potential (RMP) and whole cell input resistance (R_N_) were measured from the I-V plots built from the recordings: after identification, neurons were visualized using infrared differential interference microscopy (DIC, Nikon). Micropipettes for whole cell recordings were pulled (Sutter Instruments) from borosilicate glass tubes (1.5 mm OD, WPI) for a final DC resistance of 4–6 MΩ when filled with an internal solution with the following composition (in mM): 120 KMeSO_3_, 10 NaCl, 0.5 EGTA, 10 HEPES, 2 Mg^2+^-ATP, 0.4 Na^+^-GTP and 1% biocytin (pH = 7.2, 282 mosmol/l) for a pCa^2+^ of about 6.5 (Vilchis et al., 2000). Extracellular saline had (in mM): 124 NaCl, 2.5 KCl, 1.3 MgCl_2_, 2 CaCl_2_, 26 NaHCO_3_, 1.2 NaH_2_PO_4_ and 15 glucose (pH = 7.4, 300 mosmol/l, saturated with 95% O_2_ and 5% CO_2_). Recordings were made at room temperature (~25°C) and obtained with an Axopatch 200B amplifier (Axon Instruments) while monitored with an oscilloscope (Tektronix; de Jesús Aceves et al., 2011). Signals were filtered at 1–3 kHz and digitized at 3–9 kHz with an AT-MIO-16E4 board (National Instruments) in a PC computer. Data acquisition used custom software designed in the LabVIEW environment (National Instruments). R_N_ was measured from the slope of current-voltage (I-V plots) relationships at −60 mV. The latter were obtained with either depolarizing or hyperpolarizing rectangular voltage steps (current-clamp) or voltage commands (voltage-clamp). RMP was measured in current-clamp at zero current. Percentages of neurons exhibiting diverse signs of excitability suggesting various differentiating stages were obtained in both samples and compared (see “Results” Section).

### Toxicity Assay

Terminally differentiated MNs were exposed 20 min to vehicle or 300 μM kainate and evaluated 24 h later (Carriedo et al., 1996) by immunostaining.

### Statistical Analysis

All experiments were performed in duplicate. Results were subjected to one-way analysis of variance (ANOVA) and Bonferroni analysis or *t*-tests. For electrophysiology, the Mann-Whitney U test was used to compare unpaired samples. Multiple treatments were compared with the Kruskal-Wallis ANOVA and *post hoc* Dunn statistics. RAP, inward rectification (sag) and calcium potential differences were analyzed by Chi-square test. *P* < 0.05 was used as significance level.

## Results

### Generation of GDNF-Expressing ESC

**Figure 1:**
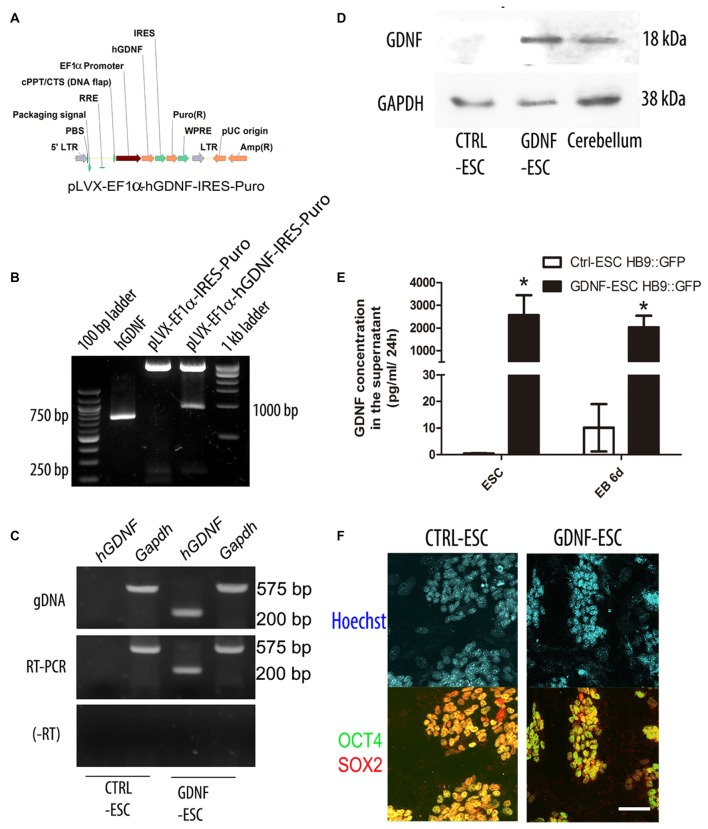
**Generation of transgenic mouse embryonic stem cells (mESC) that release glial cell-derived neurotrophic factor (GDNF) to the medium and retain pluripotency. (A)** Scheme of the EF1α::human GDNF (hGDNF) lentiviral construct. **(B)** Ligation of the *hGDNF* complementary DNA (cDNA; lane 2) into the lentiviral vector (lane 3) to produce the pLVX-EF1α-hGDNF-IRES-Puro plasmid (lane 4). **(C)** Genotyping (by endpoint PCR) and mRNA expression (by RT-PCR) after lentiviral transduction of ESC. RNA without retrotranscription (-RT). **(D)** Western blot to detect GDNF in cell lysates. Cerebellum was used as a positive control. GAPDH as a loading control **(E)** Quantification of GDNF by ELISA in conditioned media in undifferentiated ESC and differentiation embryoid bodies (EBs; white bars reflect mGDNF). *p < 0.001 vs. CTRL-ESC at the same stage. **(F)** Immunofluorescence for the pluripotency factors Sox2 and Oct4 in CTRL- and GDNF-ESC transgenic lines. Scale bar: 50 μm.

In order to produce stable ESC lines that produce hGDNF, we prepared lentiviral vectors that confers puromycin resistance (CTRL) or simultaneously allows expression of hGDNF and puromycin resistance (Figures 1A,B). Lentiviral particles were harvested and added to mESC, followed by selection with puromycin for 4 days to obtain several colonies that were picked and expanded. Genotyping was carried on using specific primers for the hGDNF cDNA that does not detect the mouse *gdnf* gene. End-point PCR confirmed that several colonies integrated the hGDNF coding sequence, and we designated these as GDNF-ESC; empty vector-transduced cells were named CTRL-ESC (Figure 1C upper panel). RT-PCR demonstrated that hGDNF mRNA is expressed in GDNF-ESC but not in CTRL-ESC (Figure 1C middle and lower panel). Western blot analysis showed that GDNF protein can be detected in cell lysates of undifferentiated GDNF-ESC, and it is absent in CTRL-ESC (Figure 1D). However, the presence of GDNF in cell lysates does not necessarily mean that the protein is being secreted in a sustained manner, especially if the cells were subject to differentiation protocols. For this reason, a well established embryoid body-based differentiation protocol was performed and samples were measured at the beginning and the end of the differentiation procedure. Conditioned media were collected and GDNF was quantified by ELISA. GDNF concentration was significantly higher in GDNF-ESC as compared to CTRL-ESC in both stages of differentiation (Figure 1E). To analyze if this increased production of GDNF altered pluripotency-associated markers, undifferentiated ESC were labeled with antibodies directed against the transcription factors Sox2 and Oct4, with similar levels found in CTRL- and GDNF-ESC, suggesting that pluripotency was not compromised by GDNF (Figure 1F). In agreement, no significant differences between CTRL-ESC and GDNF-ESC were found when the expression of the pluripotency markers *Oct4, Sox2* and* Nanog* were quantified by RT-qPCR, normalized by *Actin*: *Oct4* showed 3.14 ± 0.998 fold-increase, *Sox2* was 0.893 ± 0.137 and *Nanog 0.592 ± 0.434* in GDNF-ESC, when compared to CTRL-ESC.

### GDNF-ESC Produces More MN Precursors and Higher Numbers of Postmitotic MNs

**Figure 2:**
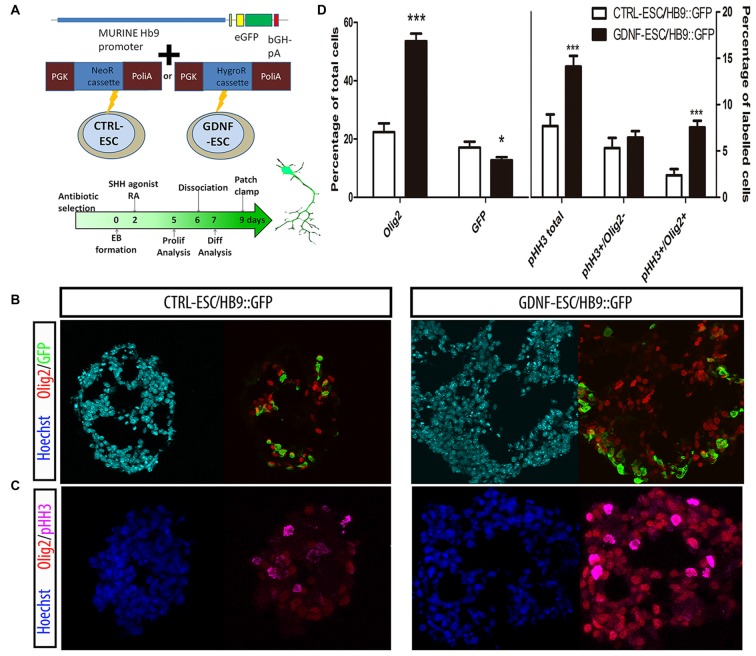
**GDNF overexpression increases the proliferation and enhances terminal differentiation to Motor neuron (MN). (A)** Scheme of the Hb9::GFP constructs inserted into CTRL- and GDNF-ESC, conferring neomycin or hygromycin B resistance, respectively, and timeline of experiments. **(B)** Representative micrographs of day 5 EBs stained to identify MN precursors (pMN; Olig2+) and MNs (GFP+). **(C)** Day 5 EBs were labeled with antibodies against Olig2 and phospho-histone H3 (pHH3), in both CTRL- and GDNF-ESC/HB9::GFP. **(D)** Quantification of experiments shown in Figure 4B (left side) and Figure 4C (right side). *p < 0.05 and ***p < 0.0001 vs. CTRL-ESC/HB9::GFP. Data from four experiments in duplicate.

GDNF is known for having several neuronal targets, which includes MNs. We decided to study MN differentiation by using a reporter construct that expresses GFP under control of the HB9 promoter, which directs its expression only in postmitotic MNs. This reporter appears early during EB formation and allows the identification of MNs. The HB9::GFP plasmid was co-transfected with either neomycin or hygromycin B resistance cassettes in CTRL- or GDNF-ESC, respectively, and selected with the appropriate antibiotic to produce CTRL-ESC/HB9::GFP or GDNF-ESC/HB9::GFP (Figure 2A). Several clones were picked and genotyped for GFP integration (data not shown). Experiments were done in six clones (three for CTRL-ESC and three for GDNF-ESC) with similar results within both groups (data not shown). We observed that during EB formation, even prior to GFP detection, GDNF-ESC EBs were larger than control, suggesting that GDNF is changing the proliferation rate during EB formation. Olig2 is a transcription factor that regulates the replication of precursors that eventually will become first MN or later on oligodendrocytes (Lee et al., 2005). At day 5 of the differentiation protocol, EBs were fixed, sliced and immunofluorescences for Olig2 and GFP were performed. GDNF-ESC/HB9::GFP EBs had significantly higher proportions of Olig2 expressing cells compared to CTRL-ESC/HB9::GFP. In parallel, a discrete but significantly lower proportion of GFP+ newborn neurons was found in GDNF-ESC/HB9:GFP vs. CTRL-ESC/HB9:GFP (Figures 2B,D). Next, we asked whether the Olig2 expansion was caused by higher proliferation rates. The phosphorylation at serine 10 of histone H3 (pHH3) is associated with mitotic activity (Hendzel et al., 1997); we observed an increased number of pHH3+ cells in GDNF-ESC/HB9::GFP cultures (Figure 2C). Olig2 and pHH3 double-positive cells were significantly increased by GDNF. The proliferative effect of GDNF seems to specifically target Olig2 positive cells, since no differences were found in pHH3+/Olig2- cells (Figures 2C,D).

**Figure 3:**
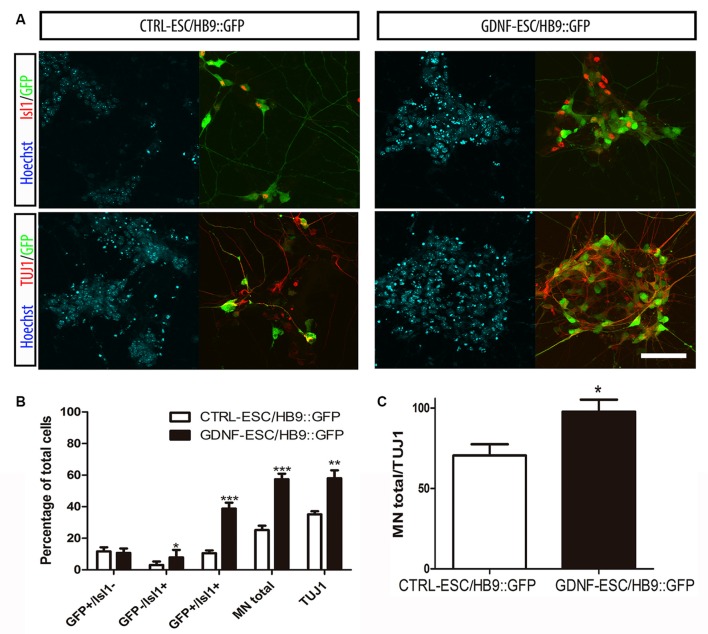
**Transgenic GDNF expression increases the yield of postmitotic MN. (A)** Immunostaining of postmitotic neurons at day 7 of differentiation. **(B)** Percentage of MNs (i.e., GFP+ or Isl1+) relative to the number of total neurons. **(C)** Percentage of MNs (i.e., GFP+ or Isl1+) relative to the number of total neurons. Scale bar: 50 μm except for Figure 4C where scale represents 47 μm. *p < 0.05, **p < 0.001 and ***p < 0.0001 vs. CTRL-ESC/HB9::GFP. Data from four experiments in duplicate.

Postmitotic neurons were evaluated after dissociation of EBs and replating. Islet1 (Isl1) is a transcription factor that is normally expressed early during MN differentiation, and together with HB9 comprise pan-MN markers (Amoroso et al., 2013). We analyzed these markers by staining for Isl1 and the HB9 reporter GFP, as well as with the TUJ1 antibody that recognize neuronal class III beta-Tubulin in differentiated cultures. We found that the GFP+/Isl1- cells did not change in GDNF-expressing cells. However, significant increases were observed for GFP-/Isl1+ and GFP+/Isl1+ cells, accounting for a 2.3-fold increase in the total MN proportion for GDNF-ESC/HB9::GFP. A less marked, albeit a significant increase (1.6-fold), was observed in young neurons labeled by TUJ1 in GDNF-cells (Figures 3A,B). Noteworthy, almost every neuron presented a MN phenotype in GDNF-ESC/HB9::GFP; since only 3% did not express GFP or Isl1, but this proportion was 30% in control cells (Figure 3C).

### Early Addition of Exogenous GDNF Increases MN Differentiation

**Figure 4:**
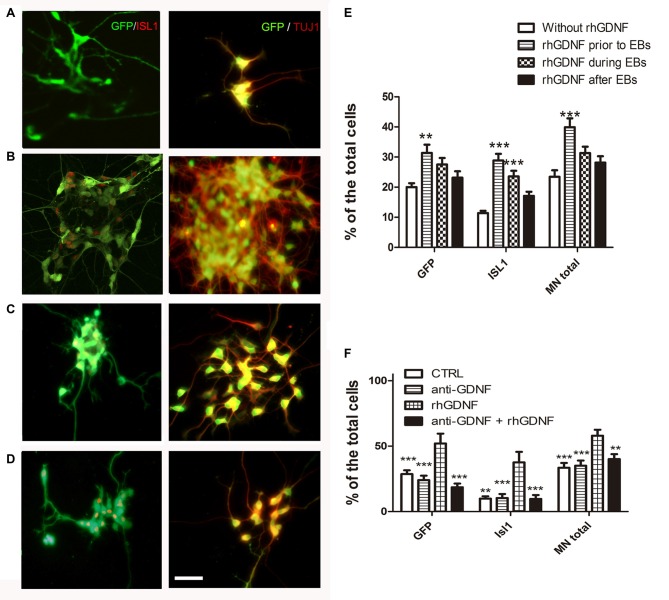
**Recombinant hGDNF (rhGDNF) exogenously added at early stages increases differentiation towards MN, which can be prevented by neutralizing anti-GDNF antibodies.** CTRL-ESC/HB9::GFP were differentiated without rhGDNF **(A)** or adding this protein only at specific periods: prior to formation of EBs **(B)**, during EBs **(C)** or after EBs **(D)**. The quantification of total MN differentiation (ISL1 and/or GFP) reveals that early and transient exposure to rhGDNF significantly increases MN induction **(E)**. **p < 0.001 and ***p < 0.0001 compared to differentiation without rhGDNF. Addition of anti-GDNF neutralizing antibodies did not affect differentiation of CTRL-ESC/HB9::GFP **(F)**. However, co-incubation of rhGDNF and anti-GDNF antibodies, significantly decreased the inductive effect of GDNF. **p < 0.001 and ***p < 0.0001 compared to differentiation with rhGDNF. Plot resulting from three independent experiments performed in duplicate. Scale bar: 50 μm.

To investigate if GDNF has to be present throughout differentiation as in the overexpressing lines, or its influence is more effective during certain stages, we transiently added recombinant hGDNF (rhGDNF) at specific periods of differentiating CTRL-ESC/HB9::GFP. These control cells were differentiated without rhGDNF (Figure 4A), or with rhGDNF added: (i) prior to formation of EBs (Figure 4B); (ii) during EBs (Figure 4C); and (iii) after dissociation and replating (Figure 4D). Although the addition of rhGDNF in these periods increased total MN differentiation relative to the condition without rhGNDF, this rise was significant only when the neurotrophin was supplemented before EB formation (Figures 4E,F). However, GDNF added solely in any of these stages, does not reach the same level of MN induction compared to the transgenic secretion (Figure 3B). The unexpected early effect of GDNF, together with the enhanced proliferation of MN precursors, suggests that GDNF effects start early-on, but the neurotrophin needs to be present throughout the differentiation time course to further increase MN differentiation as in GDNF-ESC HB9::GFP. To further validate the specificity of GDNF, we decided to incubate differentiating CTRL-ESC HB9::GFP with exogenous rhGDNF with or without neutralizing anti-GDNF (4 μg/ml) antibodies. This initial exposure was made from the pluripotent condition up to day-2 EBs. We observed that addition of anti-GDNF inhibits the increase in MN expansion promoted by exogenous rhGDNF (Figure 4F), supporting an early role of GDNF during MN differentiation.

### Overexpression of GDNF Increases Excitability and Maturation of ESC-Derived MNs

**Figure 5:**
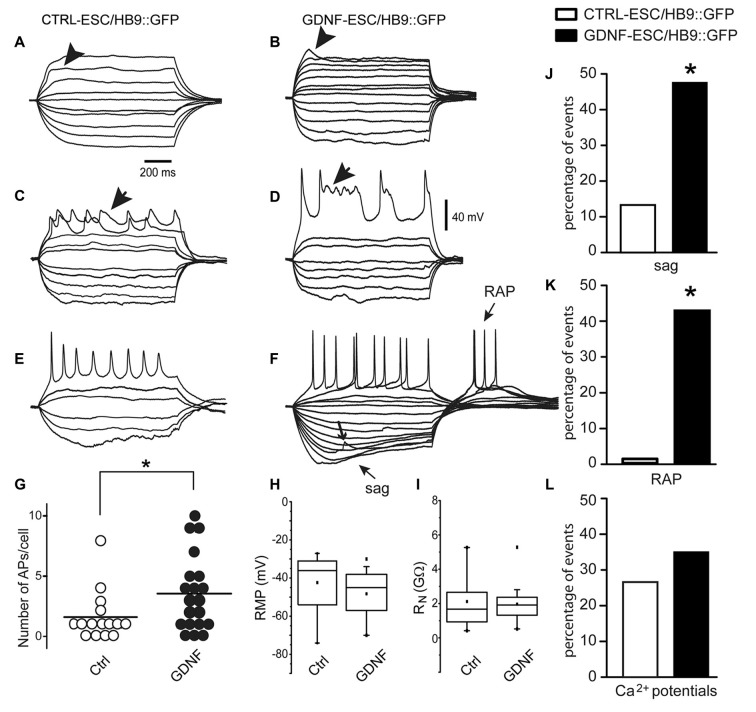
**Signs of excitability are more evident in MNs derived from GDNF-expressing cells.** At 9 days in culture, CTRL-ESC/HB9::GFP (left column) and GDNF-ESC/HB9::GFP (middle column) MNs showed various stages of differentiation. **(A–F)** show voltage responses to depolarizing and hyperpolarizing current steps (not shown) injected intracellularly. **(A,B)** Arrowheads denote local responses appearing upon mostly passive electrical properties in both classes of neurons. **(C,D)** Arrows point towards regenerative responses with mixed sodium and calcium components. **(E,F)** Repetitive firing of action potentials (APs) in both classes of neurons. GDNF MNs showed larger inward rectification denoted by depolarizing sags upon hyperpolarization and rebound APs (RAPs) upon returning from it. Arrow denotes a putative spontaneous synaptic event **(F)**. **(G)** The average number of APs, significantly larger in GDNF-expressing cells. **(H,I)** Neither resting membrane potential (RMP) nor whole neuron input resistance (R_N_) were significantly different. **(J)** Sag inward rectification was significantly larger in GDNF-expressing neurons. **(K)** RAPs after ending a hyperpolarization step were significantly higher in GDNF expressing neurons. **(L)** Ca^2+^events was not significantly different between both samples (see **C,D**). *p < 0.05.

For MNs, a reporter construct (Wichterle et al., 2002) has been designed for the prospective identification of live neurons allowing electrophysiological studies. CTRL- and GDNF-ESC were transfected with the HB9::GFP plasmid and the electrophysiological properties of four cell lines (two CTRL-ESC/HB9::GFP and two GDNF-ESC/HB9::GFP) were recorded after MN differentiation in cells identified by GFP expression. Whole-cell voltage-clamp and current-clamp recordings were made to perform current-voltage (I-V) relationships (not shown) to obtain passive membrane properties. Since our interest is focused in excitability at various stages of maturity, only voltage or currents in response to current injections or voltage commands, respectively, are depicted. Figure 5 shows that at 9 days in culture, CTRL-ESC/HB9::GFP (left column) and GDNF-ESC/HB9::GFP (right column) cells showed various stages of MN differentiation: from subthreshold non-propagating local responses (Aidley, 1998; Figure 5B, arrowhead), not equally observed in both groups (Figure 5A), mixed sodium and calcium regenerative events, known to be characteristic of MN development (Spitzer et al., 2000; Figures 5C,D, arrows) to repetitive firing of sodium-generated APs. These responses were obtained with depolarizing and hyperpolarizing current steps. GDNF-expressing MNs showed apparently larger inward rectification denoted by depolarizing sags upon hyperpolarization (sag arrow in Figure 5F) and RAPs when the hyperpolarization ended (RAP arrow; Figure 5F); the other arrow in Figure 5F denotes a putative spontaneous synaptic event. The proportion of neurons that showed firing was significantly higher in GDNF-expressing cells, showing a 2.3-fold increase compared to control MNs (Figure 5G). However, RMP was not different (Figure 5H), although 75% of the GDNF-expressing neuronal population was below the median of the control sample. Similarly, in whole neuron R_N_ no differences were noted, although the distribution of GDNF-expressing cells was compacted and with less variability (Figure 5I). Regarding more mature signs of MNs excitability (Spitzer et al., 2000; Takazawa et al., 2012), the percentage of neurons exhibiting sags (inward rectification) and RAPs were significantly larger in GDNF-expressing neurons (Figures 5J,K). Although the presence of mixed sodium and calcium regenerative events was equally seen in both samples (Figure 5L), Ca^2+^ events in GDNF-expressing neurons had longer duration and more depolarized potentials (see Figures 5C,D).

**Figure 6:**
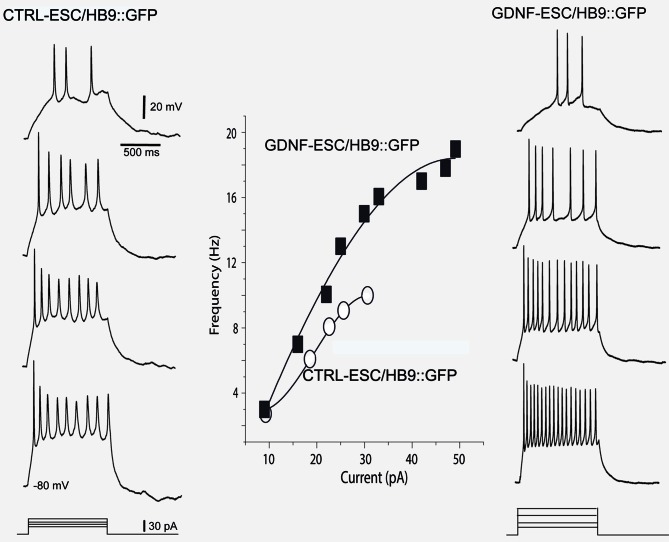
**Intensity-frequency plots show increased attained frequency in GDNF-ESC/HB9::GFP motoneurons. Left column**, from top to bottom, four representative voltage responses evoked with intracellular current injections of increasing intensity in a Ctrl-ESC/HB9::GFP neuron. Current is represented at the bottom. Frequencies attained are depicted by empty circles at the middle graph. Higher current intensities provoked a halt in firing, due to spike inactivation (not shown). **Right column**, from top to bottom, four representative voltage responses evoked with increasing intracellular current injections in a neuron producing GDNF (GDNF-ESC/HB9::GFP). Resulting frequencies are depicted by filled squares in the middle graph. Note that frequencies attained are higher in the GDNF-producing cell. Contrary to the observation with the control neuron, higher current intensities could be administered with no signs of spike inactivation in the GDNF-expressing neurons. Multiple treatments were compared with the Kruskal-Wallis analysis of variance (ANOVA) and *post hoc* Dunn statistics.

Intensity-frequency plots showed that injection of current elicited APs in both CTRL- and GDNF-ESC HB9::GFP-derived MNs. However, such frequencies were higher in GDNF-ESC/HB9::GFP MNs. Not only frequencies augmented in the GDNF-producing neurons, but higher current intensities could be administered with no signs of spike inactivation, as observed with current applications of 40 pA or more in CTRL-ESC/HB9::GFP neurons (Figure 6).

**Figure 7:**
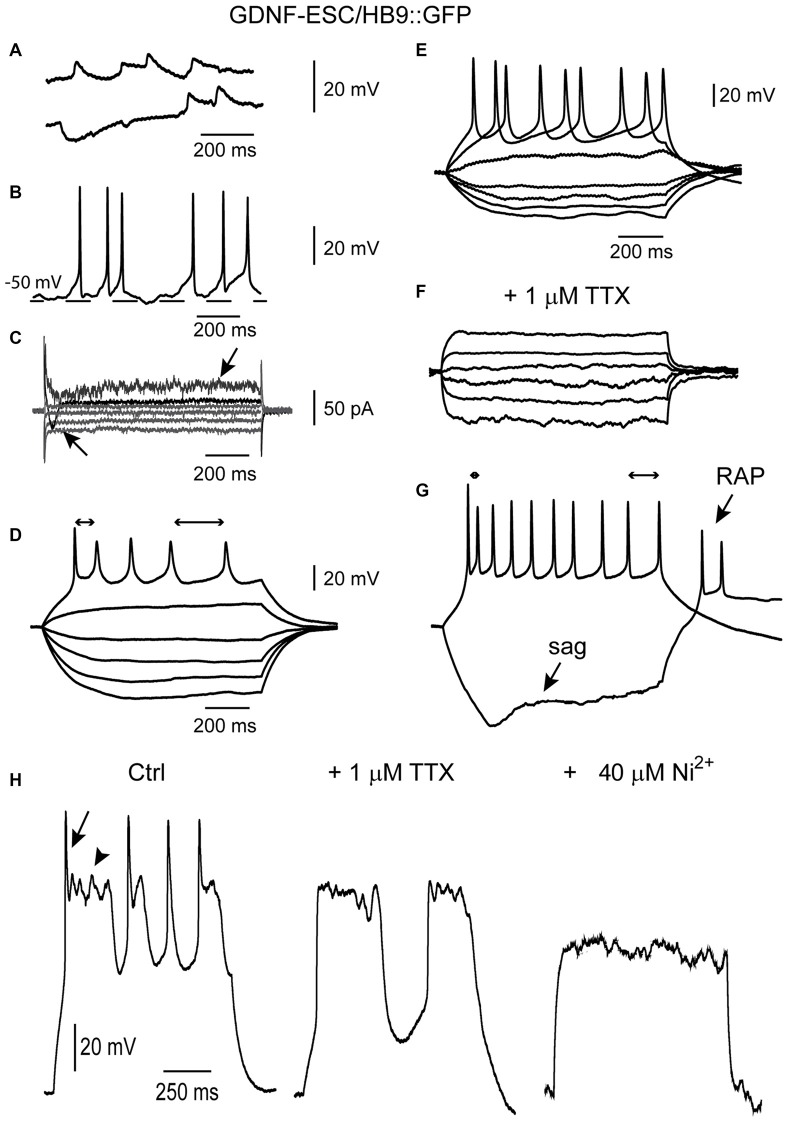
**Some maturation signs are present only in cultured GDNF-ESC/HB9::GFP MNs. (A)** Putative depolarizing and hyperpolarizing synaptic events. **(B)** At certain membrane potentials, putative synaptic events provoke the firing of APs. **(C)** Point somatic voltage clamp frequently showed inward currents (arrow pointing to top). Signs of developing delayed rectification were also observed (arrow pointing to bottom). Voltage commands are not illustrated. **(D)** Repetitive APs as well as frequency adaptation (bidirectional arrows show different interspike intervals at the beginning and end of the evoked spike train). **(E,F)** Repetitive APs **(E)** are blocked **(F)** by 1 μM tetrodotoxin (TTX). **(G)** A more mature MN showing brief repetitive APs exhibiting frequency adaptation (bidirectional arrows), the sag of inward rectification (bottom arrow) and RAPs (top arrow) at the end of the hyperpolarization. **(H)** Control (left) recording showing mixed regenerative and repetitive propagating events in one neuron evoked with intracellular current steps (not shown). Arrow, a putative Na^+^ current; arrowhead: a Ca^2+^ plateau. After adding 1 μM TTX, Na^+^ spikes were gone, leaving the Ca^2+^ potentials (middle). Finally, after adding 40 μM Ni^2+^ to the same cell in the continuous presence of TTX, Ca^2+^ potentials were also blocked (right).

Figure 7 illustrates some signs of excitability solely present in the GDNF-ESC/HB9::GFP cells, such as a spontaneous putative depolarizing and hyperpolarizing synaptic potentials (Figure 7A), that were abolished by the glutamate receptor antagonist CNQX (data not shown), spontaneous firing of APs (Figure 7B), inward and outward voltage-gated currents during voltage-clamp recordings (Figure 7C). When cells presented repetitive firing, it was common to observe SFA: first interspike interval was shorter than the last in the train (double-headed arrows in Figure 7D). Moreover, when repetitive firing could be evoked, it was completely blocked by 1 μM of TTX (Figures 7E,F, same cell), suggesting that APs were generated by sodium currents. APs exhibited SFA, sag inward rectification and RAPs (Figure 7G). Cells in intermediate stages showed mixed sodium and calcium regenerative events (Spitzer et al., 2000; see also Figures 5C,D): TTX blocked the fast sodium component (Figure 7H left to middle), while 40 μM Ni^2+^ (or Co^2+^, not shown) blocked the slower plateau-like calcium component (Figure 7H middle to right). After TTX and Ni^2+^, stronger current injections could not evoke any signs of excitability.

### GDNF-ESC-Derived MNs are More Resistant to Kainic Acid

**Figure 8:**
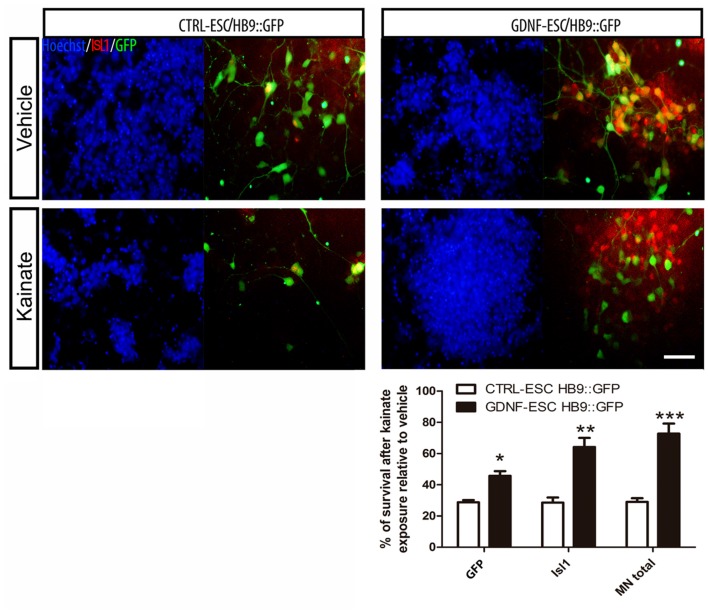
**ESC-derived MNs exposed to kainate are protected when GDNF is overexpressed.** Differentiated cultures were exposed to vehicle or kainate and assessed survival. The quantification of GFP+, Isl1+ or total MNs (i.e., GFP+ or Isl1+) is presented in the plot. Average ± SEM from three independent experiments performed by duplicate. Scale bar: 50 μm. *p < 0.05, **p < 0.001 and ***p < 0.0001 vs. CTRL-ESC/HB9::GFP.

MNs are susceptible to a wide variety of insults including oxidative stress (Kruman et al., 1999), energy depletion (Guo and Bhat, 2007) and excitotoxicity (Foran and Trotti, 2009). Glutamate or its agonists AMPA or kainate have been used to induce MN degeneration. MNs were incubated with kainate and the number of cells expressing GFP or ISL1 counted. Control MNs have an average reduction of 71% whereas GDNF-expressing cells have a 27% decrease. The survival of GFP+, ISL1+ and total MNs was significantly higher in GDNF-ESC/HB9::GFP compared to CTRL-ESC/HB9:GFP (Figure 8).

## Discussion

In this article, mESC lines with sustained GDNF-secreting properties were developed. This approach allowed us to: (1) discover that GDNF in EBs induced higher proliferation resulting in more committed MN precursors (pMN); (2) report that GDNF induced electrical maturation in MNs in the absence of exogenous factors; and (3) observe self-protection against injury in MN cultures. Further, the addition of exogenous rhGDNF demonstrates that this trophic factor enhances MN differentiation when added at the pluripotent state.

The GDNF-overexpressing ESC lines were capable of robust MN differentiation, but moreover, pluripotency-associated factors such as Oct4 and Sox2 were not altered suggesting that stemness is intact. In differentiation protocols of pluripotent stem cells towards MNs, GDNF is commonly included as a trophic factor for postmitotic MNs. However, whether neural precursors are responsive to GDNF remained an open question. In such case, GDNF would not only impact neuronal survival, but also enhances cell commitment. Our findings suggest that GDNF might be directing the commitment of neurons towards specific phenotypes and not only increasing the survival of neurons; the same question might be addressed for other neurotrophic factors important for MN differentiation or survival such as VEGF.

GDNF and its receptors are expressed in proliferating zones during spinal cord development, and thus GDNF might have additional roles to neuronal survival (Trupp et al., 1995; Treanor et al., 1996; Golden et al., 1999; Homma et al., 2000). Here, we describe an increase in proliferation that is specific for pMN in GDNF-ESC/HB9::GFP cultures. OLIG2 maintains the pMN cycling by antagonizing the neuronal-promoting action of NGN2; this inhibition abolishes the expression of neuronal genes, including MN transcription factors such as HB9 (Lee et al., 2005). In agreement, a decrease in GFP+ cells was observed at day 5 of EBs in GDNF-ESC/HB9::GFP. A recent study has shown that a cocktail of neurotrophic factors, including GDNF increases the presence of BrdU+ cells, thus raising the total number of MN, but without altering the percentage of differentiated MN from hESC (Lamas et al., 2014). The intriguing observation that exogenous GDNF added transiently in pluripotent ESC enhanced terminal MN differentiation should stimulate future explorations on the mechanisms triggered by this trophic factor in undifferentiated cells. Neutralizing antibodies against GDNF abolished the positive MN differentiating effect of soluble rhGDNF, which points to a membrane-bound receptor-mediated action.

Regarding MN differentiation, in our experiments GDNF increases the number of MNs, although some subpopulations seem to be favored because GFP-/ISL1+ and GFP+/ISL1+ are augmented relative to control cells. The significant rise in TUJ1+ cells might be due to pMN expansion in the presence of GDNF. In GDNF-ESC/HB9::GFP cultures, practically all neurons are MNs, which can be explained by at least two reasons: (1) GDNF specifies the differentiating cells to become MNs; for instance, GDNF is necessary for the specification of neurons in the dorsal root ganglia (Ernsberger, 2008); or (2) GDNF non-responsive neurons die, leaving a near-total enrichment of MNs in culture; both possibilities are not mutually exclusive. *In vivo*, not all MNs respond equally to alterations in GDNF signaling. *Gdnf* KO mice present diminished numbers of spinal and facial nuclei MNs (Moore et al., 1996; Pichel et al., 1996). Thus, *Gdnf* KO studies have shown that despite the importance of this gene in the development of motor and other types of neurons, it is not absolutely required for CNS development, since the lack of this protein does not cause major defects; however, the plethora of morphogens and trophic factors switching on and off along development, as well as the spatial constraints in the course of neuronal development, allow the survival of different types MNs in *Gdnf* mutants. Conversely, muscle-specific overexpression of GDNF alters neuron counts and neuromuscular junctions, but not in all nuclei (Buss et al., 2006).

Recently, mESC lines that constitutively express GDNF were reported. In this work (Bryson et al., 2014), mESC engineered to express GFP (under the *HB9* promoter), channelrhodopsin-2 and GDNF were differentiated to MNs. Notably, MNs expressing GDNF survived longer than the parental cell line. GDNF MNs co-cultured with ESC-derived astrocytes for 7 days were not able to sustain a high frequency of APs after electrical stimulation, similar to MNs derived from CTRL-ESC/HB9::GFP. Only MNs co-cultured with glia for 21–35 days showed a sustained response of APs frequency with increasing depolarizing current, similar to the 9 day-old MNs differentiated from our GDNF-ESC/HB9::GFP, which did not require astrocytes. Such dissimilarities might be explained by the different methods used to produce the transgenic ESC: electroporation (Bryson et al., 2014) vs. lentivirus, and/or the promoter driving *GDNF*: CAG (Bryson et al., 2014) vs. EF1α. In these previous studies, GDNF does not seem to interfere with the pluripotency program, since their cell lines produced MN and astrocytes (Bryson et al., 2014); we now show that Sox2 and Oct4, important transcription factors associated to the pluripotent state, are expressed GDNF-ESC and CTRL-ESC, previous to differentiation.

Electrophysiological recordings at 9 days *in vitro* showed diverse signs of excitability. Cells with subthreshold local responses (Aidley, 1998) to more mature cells exhibiting repetitive firing, SFA, sag inward rectification and RAPs, when leaving a strong hyperpolarization, were observed (Takazawa et al., 2012). Several of these signs were more pronounced in the GDNF-expressing clones. Recently, fibroblasts were treated with small molecules to directly convert to glutamatergic and GABAergic neurons (Li et al., 2015). Plating of these newborn neurons on astrocytes enhanced functional properties of the membrane. Primary glial or neuronal cells promoted the appearance of spontaneous excitatory postsynaptic currents in about 50% of neurons and produced narrower APs and SFA in the recorded chemically-induced neurons (Li et al., 2015); these electrophysiological features have been associated with maturation during neural development (Spitzer et al., 2000). It is noteworthy that, our GDNF-secreting cells achieved similar maturation, but in the absence of primary astrocytes.

It has been shown that GDNF can modulate acute and chronic changes in excitability via ion channel regulation. In mesencephalic dopamine neurons, GDNF induces inactivation of potassium A currents and increase excitability through a MAP kinase-dependent pathway (Yang et al., 2001). In enteric neurons, GDNF inhibits the delayed inward rectifying potassium current (Zeng et al., 2010). After a nerve section, GDNF treatment promotes a higher level of mRNA of sodium channels correlated with higher conductance in neurons from the dorsal root ganglia (Cummins et al., 2000). In our experiments, GDNF increased MNs excitability and the appearance of other signs of maturation; further studies should establish if similar GDNF-triggered mechanisms are present in ESC-derived MNs.

We believe that our GDNF-ESC lines provide two primary benefits: they exhibit enhanced neuronal differentiation/maturation and if grafted, might provide a constant GDNF delivery by autocrine secretion. However, a note of caution is appropriate, since other work has shown that gene delivery of *GDNF* with lentiviral vectors, applied to rats with the sciatic nerve section, induces the initial extension of axons towards the source of GDNF. Later, the same motor axons become entrapped (“the candy-store effect”) by local GDNF overexpression, preventing its crossing through the injury site to reach the distal portion, which results in aggravation of the condition, as assessed both morphologically and functionally (Tannemaat et al., 2008; Hoyng et al., 2014). Grafting of these cells is a possible application, because self-secretion of GDNF to MN might enhance its survival after transplantation. In fact, GDNF-expressing MN were grafted in a model of sciatic nerve injury and showed to survive up to 35 days promoting reinnervation (Bryson et al., 2014).

GDNF was first characterized as a protecting molecule for dopaminergic neurons (Lin et al., 1993). *In vitro*, this neuroprotection has been observed when GDNF is added to the media (Meyer et al., 2001), or when co-cultures of GDNF-secreting cells are performed. The *in vivo* administration of GDNF is more complicated and includes perfusion with pumps, multiple injections of virus or co-grafts with other GDNF-secreting cells (Akerud et al., 2001; Deierborg et al., 2008). Neurons that secrete GDNF might have higher survival rates after grafting in animal models of neurodegenerative diseases.

## Author Contributions

DC participated in conception and design, collection and/or assembly or data, data analysis and interpretation, manuscript writing, final approval of manuscript. YR-A, RH-M, and IE-A: collection and/or assembly or data, final approval of manuscript. JB: data analysis and interpretation, manuscript writing, final approval of manuscript. IV: financial support, data analysis and interpretation, manuscript writing, final approval of manuscript.

## Funding

This work was supported by grants from Consejo Nacional de Ciencia y Tecnología (CONACyT CB09/131281 and Red Temática Células Troncales y Medicina Regenerativa) to IV and Dirección General de Asuntos del Personal Académico, Universidad Nacional Autónoma de México (Papiit IN208713 and IN213716 to IV and IN202814 to JB). DC, YR-A, RH-M and IE-A received graduate fellowships from CONACyT and data in this work is part of DC doctoral dissertation in the Posgrado en Ciencias Bioquímicas de la Universidad Nacional Autónoma de México.

## Conflict of Interest Statement

The authors declare that the research was conducted in the absence of any commercial or financial relationships that could be construed as a potential conflict of interest.
